# ICD-related risk model predicts the prognosis and immunotherapy response of patients with liver cancer

**DOI:** 10.3389/fphar.2023.1202823

**Published:** 2023-06-08

**Authors:** Duntao Su, Zeyu Zhang, Fada Xia, Qiuju Liang, Yuanhong Liu, Wei Liu, Zhijie Xu

**Affiliations:** ^1^ Department of General Surgery, Xiangya Hospital, Central South University, Changsha, Hunan, China; ^2^ National Clinical Research Center for Geriatric Disorders, Xiangya Hospital, Central South University, Changsha, Hunan, China; ^3^ Department of Pharmacy, Xiangya Hospital, Central South University, Changsha, Hunan, China; ^4^ Department of Pathology, Xiangya Hospital, Central South University, Changsha, Hunan, China

**Keywords:** liver cancer, immunogenic cell death, prognosis, signature, tumor immune microenvironment

## Abstract

Immunogenic cell death (ICD) is a novel cell death mechanism that activates and regulates the immune system against cancer. However, its prognostic value in liver cancer remains unclear. Here, several algorithms such as correlation analysis, Cox regression analysis, and Lasso regression analysis were carried out to evaluate the prognostic value of ICD-related genes in patients with liver cancer. Three ICD-related prognostic genes, the prion protein gene (PRNP), dynamin 1-like gene (DNM1L), and caspase-8 (CASP8), were identified and used to construct a risk signature. Patients with liver cancer were categorized into high- and low-risk groups using the ICD-related signature. Subsequently, a multivariate regression analysis revealed that the signature was an independent risk factor in liver cancer [hazard ratio (HR) = 6.839; 95% confidence interval (CI) = 1.625–78.785]. Patient survival was also predicted using the risk model, with area under the curve values of 0.75, 0.70, and 0.69 for 1-, 3-, and 5-year survival, respectively. Finally, a prognostic nomogram containing the clinical characteristics and risk scores of patients was constructed. The constructed ICD-related signature could serve as a prognostic and immunotherapeutic biomarker in liver cancer.

## Introduction

Liver cancer is extremely malignant, and its onset is frequently concealed, with most patients being diagnosed at a late stage. The fatality rate from liver cell cancer has risen steadily over the last few decades (Xu et al., 2022). With the advancement of medical technology, the emergence of novel approaches such as targeted therapy and immunotherapy has considerably increased the survival time of patients with liver cancer (Kalasekar et al., 2021). Sorafenib, for example, remains the only medicine approved for the systemic treatment of advanced hepatocellular carcinoma (HCC), but its efficacy is limited (Li et al., 2021; Wu et al., 2022). As a result, new biological indicators and prediction models are required to accurately predict the immunotherapy response of patients with liver cancer.

Immunogenic cell death (ICD) is a new cell death mechanism that involves the activation and regulation of the immune system against cancer (Zhang et al., 2021). During ICD, dead cells release various substances and antigens to interact with antigen-presenting cells or other immune cells. These immunogenic molecules are called damage-associated molecular patterns (DAMPs). ICD kills cancer cells by triggering specific tumor immune responses (Kim et al., 2021). Notably, Food and Drug Administration-approved ICD-based drugs have been used in treating melanoma and small-cell lung cancer (Markham, 2020; Tzogani et al., 2021). A study demonstrated that disulfiram and copper can synergistically induce ICD in HCC cells by promoting dendritic cell maturation and CD8^+^ T cell cytotoxicity (Gao et al., 2022). However, there are currently few studies on the prognostic and therapeutic value of ICD signaling in patients with liver cancer. Moreover, a deeper comprehension and investigation of ICD-related molecules can yield novel perspectives and insights regarding the occurrence, treatment, and prognosis of liver cancer.

In this study, we constructed a risk model for liver cancer prognosis based on the differential expression of ICD-related genes. Based on the median cut-off risk score, we divided samples into high-risk and low-risk groups. We also evaluated the risk model’s prognostic prediction capacity using an external cohort. The immune status of the two groups was then assessed. Finally, we combined clinicopathological variables with risk score to develop an effective nomogram for predicting samples survival rates. The detailed flowchart can be seen in Supplementary Figure S1. This model could be useful for predicting the immunotherapy response of patients with liver cancer.

## Materials and methods

### Identification of ICD-related genes

The Cancer Genome Atlas (TCGA, http://cancergenome.nih.gov/), the University of California Santa Cruz (UCSC, http://xena.ucsc.edu), and Xena Browser (TCGA database version: Data Release 31.0, 29 October 2021) served as the primary sources of patients’ information in this study. Patients with complete clinical and survival information were included in the study, whereas those with incomplete information were excluded. A total of 39 normal samples and 377 liver cancer samples in the TCGA-HCC datasets were extracted from UCSC databases. The GSE65372 (Zhao et al., 2021a) and GSE25097 (Sung et al., 2012) from Gene Expression Omnibus (GEO, https://www.ncbi.nlm.nih.gov/go/) were used to screen the different genes associated with liver cancer. In addition, 138 ICD-related genes were acquired from Zhang’s report (Zhang and Chen, 2022). The overlap of differentially expressed ICD-related genes in TCGA, GSE65372, and GSE25097 datasets was identified.

### Construction of the prognostic signature based on ICD-related genes

Lasso regression analysis was performed to select the prognostic ICD-related genes. The risk score was calculated using the formula: risk score = expression of (ICD-related genes 1) × (β1 of ICD-related genes 1) + expression of (ICD-related genes 2) × (β2 of ICD-related genes 2) + expression of (ICD-related genes 3) × (β3 of ICD-related genes 3) (Tibshirani, 1997). To evaluate the diagnostic and predictive value of the signature, the “survminer” and “TimeROC” R packages were used to plot the receiver operating characteristic (ROC) and the Kaplan-Meier curves (Blanche et al., 2013). Univariate and multivariate regression analyses were also used to verify the predictive value of this risk model. The nomogram for estimating the 1-, 3-, and 5-year survival probability of patients with liver cancer was constructed using the “rms” R package. An alluvial plot was used to confirm the predictive value of the signature in patients with clinical and pathological characteristics of liver cancer. In addition, decision curve analysis (DCA) was performed using the “rmda” R package to confirm the clinical significance of the signature.

### Immune analysis

To evaluate the effectiveness of immunotherapy, immune checkpoint blockade was predicted using ImmuCellAI (http://bioinfo.life.hust.edu.cn/ImmuCellAI#!/) (Miao et al., 2020). The tumor purity and proportion of infiltrating stromal/immune cells in the high- and low-risk groups were also determined using the CIBERSOFT (Newman et al., 2015) and TIMER (Li et al., 2016) methods.

### Gene set enrichment and functional enrichment analyses

Kyoto Encyclopedia of Genes and Genomes (KEGG) (https://metascape.org/gp/index.html#/main/step1) and Gene Ontology (GO) (https://proteomaps.net/) (Subramanian et al., 2005) were used for signaling pathway enrichment and functional annotation analyses, respectively.

### Statistical analysis

All statistical evaluations were performed using the R software (version 4.0.1). Nonparametric tests and one-way analysis of variance were used when necessary. Statistical significance was defined as a *p*-value < 0.05.

## Results

### Differential expression and gene ontology analysis of ICD-related genes

**FIGURE 1:**
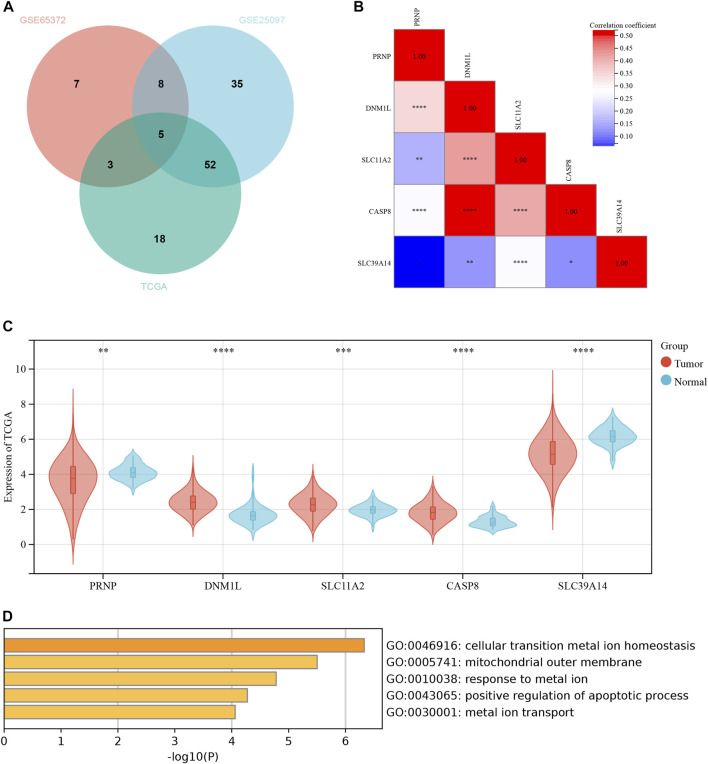
Differential expression of ICD-related genes between normal and liver cancer tissues. **(A)** The Venn diagram in GSE65372, GSE25097 and TCGA. **(B)** The heatmap of 5 ICD-related genes in normal and liver cancer tissues. **(C)** The expression of 5 ICD-related genes in normal and liver cancer tissues. **(D)** The GO functional analyses in the 5 ICD-related genes. *p < 0.05; **p < 0.01; ***p < 0.001; ****p < 0.0001.

Due to the lack of additional datasets containing survival information, we included multiple GEO datasets for the analysis of differential genes to improve the robustness of the study findings. We conducted a differential analysis of ICD-related genes using datasets from three databases: GEO65372, GEO25097, and TCGA-HCC. The results were further intersected, and the resulting Venn diagram is shown in Figure 1A. Supplementary Table S1; Figure 1A present detailed information for the Venn diagram. Five ICD-related genes were preliminarily identified. Next, we performed a correlation analysis and found that the genes CASP8 and DNM1L had the strongest correlation (Figure 1B). The results of differential expression between tumor and normal tissues are shown in Figure 1C. In the TCGA database, all five genes exhibited significant differences. The GO enrichment analysis indicated that these differentially expressed genes were mainly enriched in cellular transition metal ion homeostasis (Figure 1D).

### Establishment and verification of the ICD-related risk signature

**FIGURE 2:**
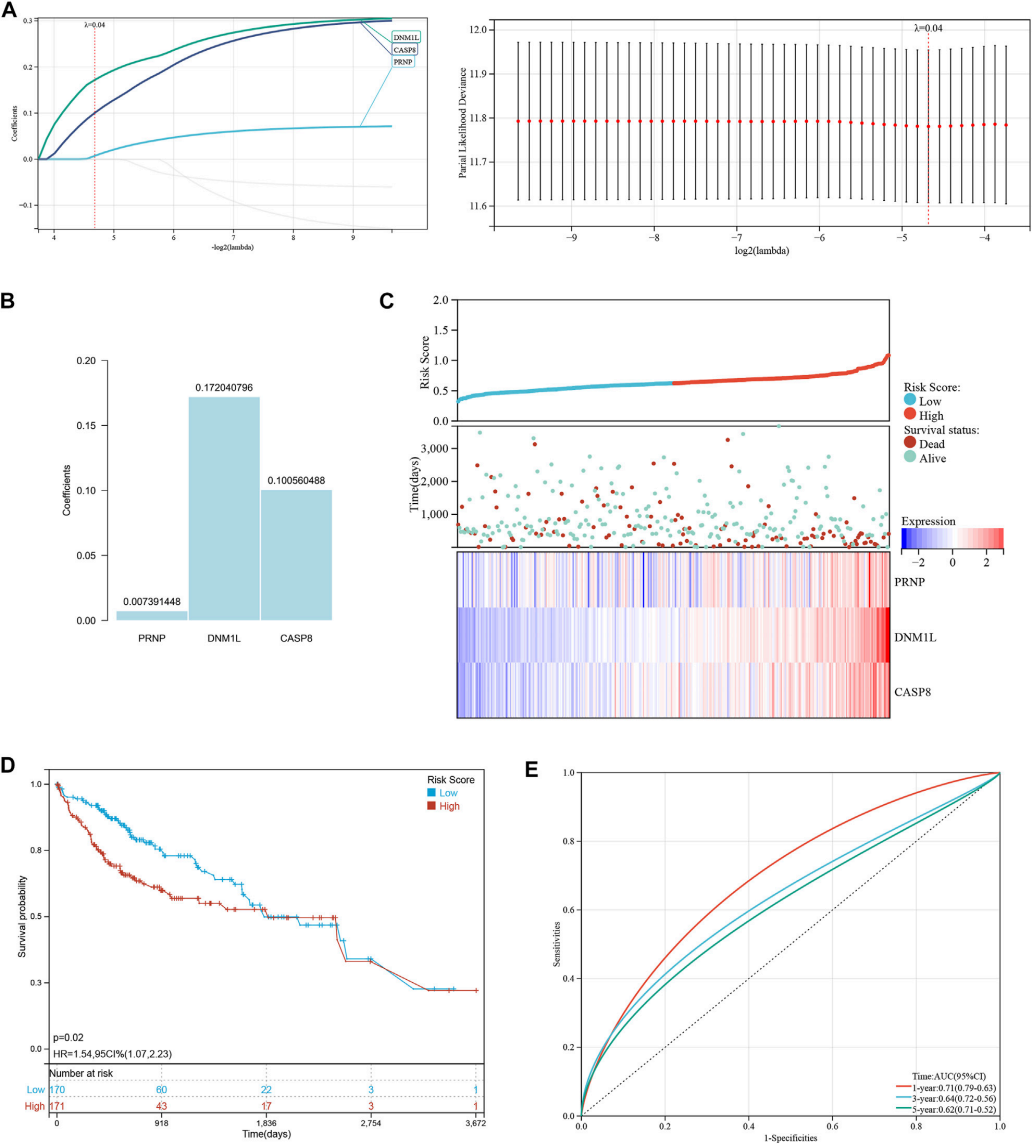
Establishment and verification of a risk signature related to ICD-related genes. **(A, B)** The Lasso-Cox regression analysis in ICD-related genes. **(C)** Distribution of risk scores and overall survival status. **(D)** Kaplan-Meier curves for the overall survival of patients in the high- and low-risk groups. **(E)** The time-dependent ROC curves supporting prognostic accuracy of the risk score.

Lasso-Cox regression analysis was used to further analyze the differentially expressed genes, and three genes, PRNP, DNM1L, and CASP8 were found to be statistically significant enough to be used to construct a risk model. The results of the Lasso-Cox regression analysis were also used to generate the risk score for each sample. Because the λ value was 0.04, the three ICD-related genes were used to build a risk model. The risk score was calculated as follows: (PRNP) * (0.007391448) + (DNM1L) * (0.172040796) + (CASP8) * (0.100560488) (Figures 2A, B). The distribution of risk scores and overall survival status (Figure 2C) and Kaplan-Meier curve (Figure 2D) demonstrated that the prognosis of patients in the high-risk group was poorer than that of those in the low-risk group. Table 1 displays the clinicopathological characteristics of patients in the high- and low-risk groups. The ROC curve revealed that the risk score had a strong predictive ability, with area under the curve values of 0.71, 0.66, and 0.61 for predicting 1-, 3-, and 5-year survival, respectively (Figure 2E).

**TABLE1:** Associations between the signature and patient characteristics in high and low risk groups cohort.

Characteristics	High (N = 170)	Low (N = 171)	Total (N = 341)	*p-*value
Age				
Mean ± SD	57.78 ± 14.33	60.40 ± 12.08	59.10 ± 13.30	
Median [min-max]	59.00 [16.00,85.00]	62.00 [20.00,82.00]	61.00 [16.00,85.00]	
gender				0.02
female	64 (18.77%)	44 (12.90%)	108 (31.67%)	
Male	106 (31.09%)	127 (37.24%)	233 (68.33%)	
tumor_stage				0.01
stage i	76 (22.29%)	94 (27.57%)	170 (49.85%)	
stage ii	42 (12.32%)	42 (12.32%)	84 (24.63%)	
stage iii	52 (15.25%)	31 (9.09%)	83 (24.34%)	
stage iv	0 (0.0e+0%)	4 (1.17%)	4 (1.17%)	
pathologic_M				0.25
M0	170 (49.85%)	168 (49.27%)	338 (99.12%)	
M1	0 (0.0e+0%)	3 (0.88%)	3 (0.88%)	
pathologic_N				0.61
N0	167 (48.97%)	170 (49.85%)	337 (98.83%)	
N1	3 (0.88%)	1 (0.29%)	4 (1.17%)	
pathologic_T				0.1
T1+T2	120 (35.19%)	137 (40.18%)	257 (75.37%)	
T3	45 (13.20%)	29 (8.50%)	74 (21.70%)	
T4	5 (1.47%)	5 (1.47%)	10 (2.93%)	

**FIGURE 3:**
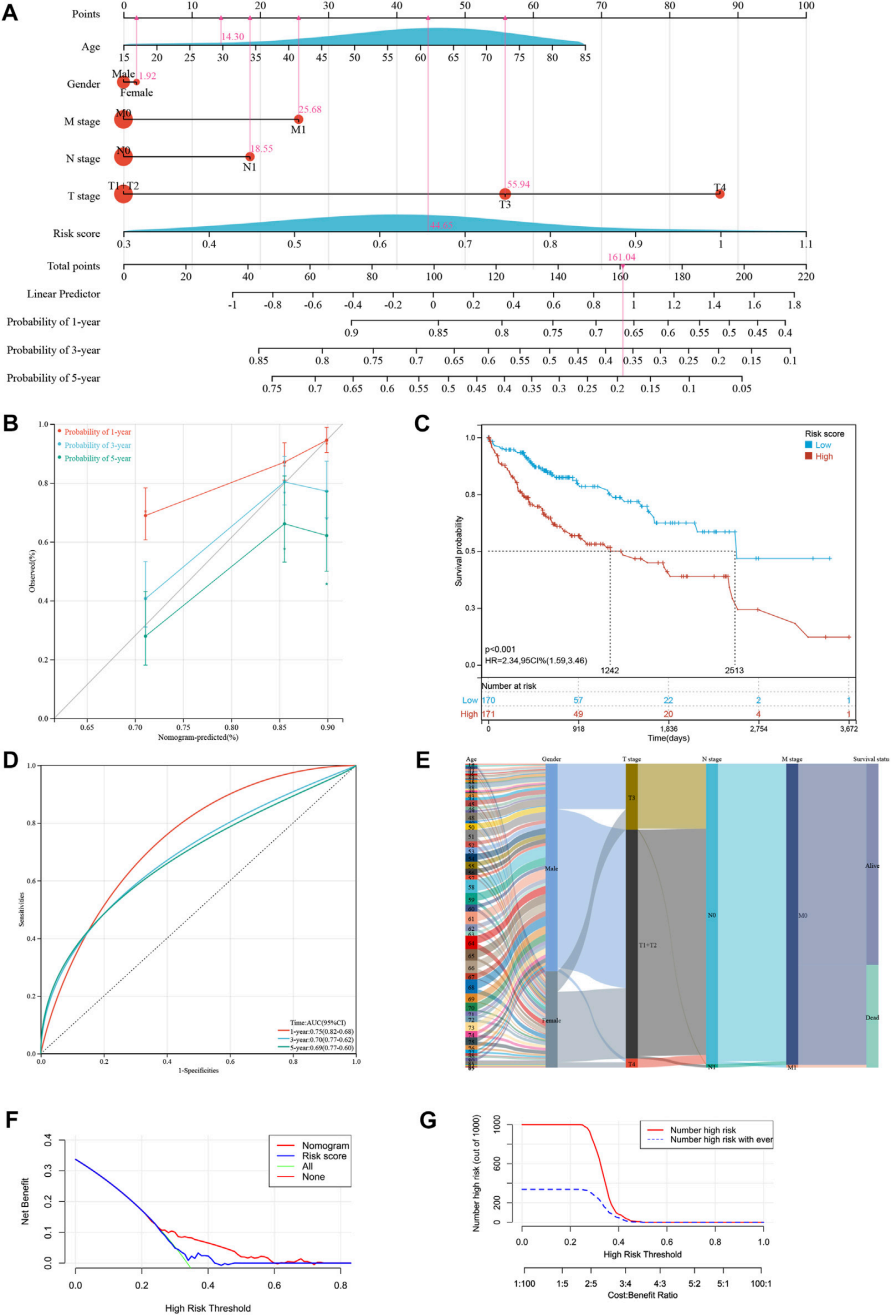
Developing a new nomogram with clinicopathological information **(A)** The nomogram of the risk model. **(B)** The calibration curves of the nomogram of risk model. **(C)** Kaplan-Meier curves for the overall survival of patients in the high- and low-risk groups based on risk model. **(D)** The time-dependent ROC curves supporting prognostic accuracy of the risk score based on risk model. **(E)** Sankey diagram showing the connection degree between the clinicopathological information and survival status. **(F)** The DCA curves of clinical practicability of the nomogram. **(G)** The nomogram provided greater net benefit (NB) than a conventional single clinicopathological characteristic.

To increase the application of the risk model, clinicopathological patient data and risk scores were integrated to construct a nomogram. The multivariate and univariate Cox regression analyses revealed the M stage, T stage, and risk scores were significantly related to prognosis (Table 2). After merging these parameters, a nomogram was developed, and scores were awarded to each patient (Figure 3A **)**. For example, the clinical information of a patient with HCC was stage M1, T3, approximately 30 years of age, and female. Including the risk score, this patient’s overall score was 161.04. Figure 3A depicts the 1-, 3-, and 5-year patient survival rates. The calibration curves showed that the nomogram accurately predicted the prognosis of the patients (Figure 3B). In addition, Figure 3C depicts the 1-, 3-, and 5-year OS of the patients (HR = 2.34, 95% CI = 1.59–3.46). Figure 3D presents the ROC curve for the nomogram. A high score signified an unfavorable prognosis. Figure 3E depicts the prognosis of patients combined with various influencing factors. DCA curves (Figure 3F) further confirmed the nomogram’s clinical applicability. The nomogram provided greater net benefit than a conventional single clinicopathological characteristic (Figure 3G).

**TABLE2:** Univariate and multivariate analyses of risk factors in the cohort.

Variables	Univariate analysis		Multivariate analysis	
	HR (95% CI)	*p*-value	HR (95% CI)	*p*-value
Age (years)	1.011 (0.997–1.026)	0.134		
Gender				
Female	1 (ref)			
Male	0.753 (0.517 1.096)	0.138		
T stage				
T1+T2	1 (ref)			
T3	2.357 (1.592 3.490)	<0.001	2.271 (1.531 3.367)	<0.001
T4	4.675 (2.124 10.290)	<0.001	3.881 (1.459 10.328)	0.007
N stage				
N0	1 (ref)			
N1	2.008 (0.494 8.161)	0.330		
M stage				
M0	1 (ref)			
M1	3.894 (1.231 12.320)	0.021	1.536 (0.364 6.486)	0.559
Risk score	8.130 (1.982 33.360)	0.004	6.839 (1.625 28.784)	0.009

### The relationship between the ICD-related risk signature and tumor microenvironment

**FIGURE 4:**
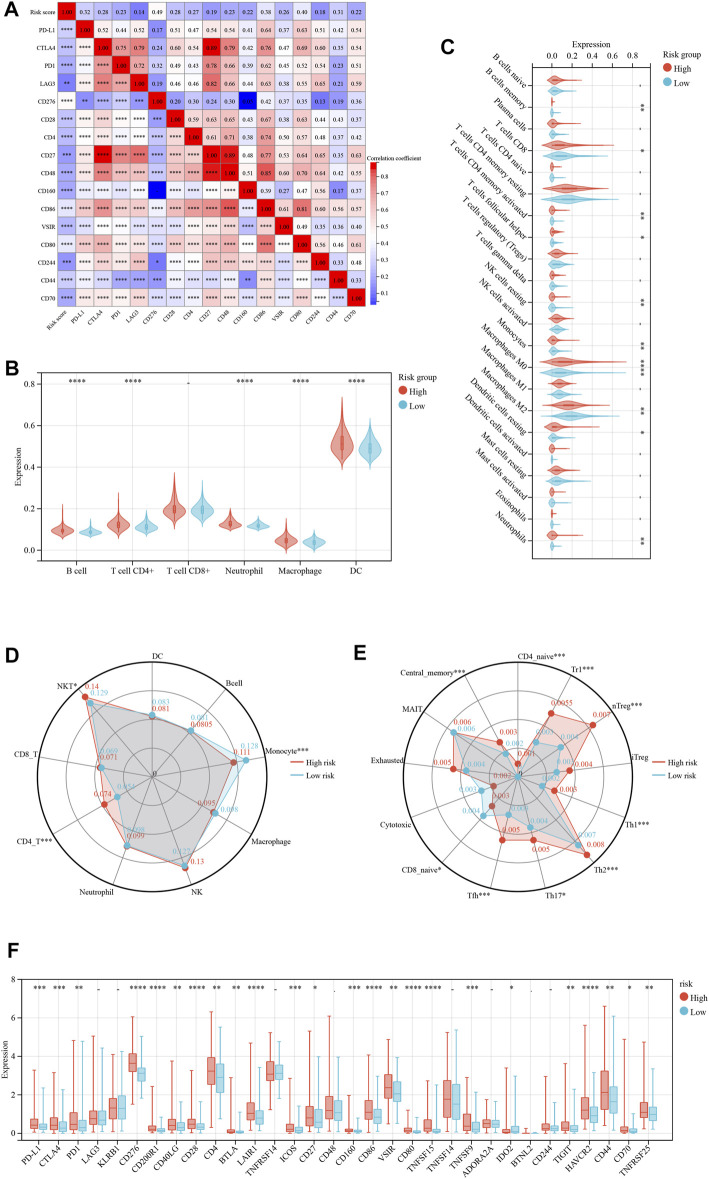
Risk model based on immune cell infiltration and clinicopathological factors. **(A)** The correlation hot map between immune check point and risk score. **(B)** The TIMER scores between the low- and high-risk immune cell group. **(C)** The CIBERSOFT scores between the low- and high-risk immune cell group. **(D, E)** The immune cell infiltration between the low- and high-risk group. **(F)** The immune check point of difference expresses between the low- and high-risk group. *p < 0.05; **p < 0.01; ***p < 0.001; ****p < 0.0001.

Immunological ICD treatments have yielded favorable clinical outcomes in recent years (Zhu et al., 2020; Dong et al., 2022). Resultantly, we analyzed immunotherapy checkpoints in the high- and low-risk groups. Figure 4A depicts the correlation between various immunotherapy checkpoints. The link between CD27 and CD48 was the strongest, with a correlation coefficient of 0.89. We analyzed the immune states using several immune scoring methods, such as the TIMER (Figure 4B) and CIBERSOFT algorithm (Figure 4C). The results showed that the expression of multiple immune cells was significantly different between the high- and low-risk groups. Then, we found significant differences in the infiltration of monocytes, CD4-T cells, and NKT **(Natural killer T)** cells between the high- and low-risk groups (Figure 4D). Similarly, we discovered that there were significant differences in the infiltration of CD4 naive, Trl, nTreg, Th1, Th2, Th17, Tfh, CD8 naive, and central memory cells (Figure 4E). By studying the expression of immunotherapy checkpoints in the patients (Figure 4F **)**, we discovered that the majority of immunotherapy checkpoints were significantly expressed at higher levels in the high-risk group than in the low-risk group, suggesting that the patients in the high-risk group might be more responsive to immunotherapy checkpoint-based therapies.

### Biological pathways associated with ICD-related genes

**FIGURE 5:**
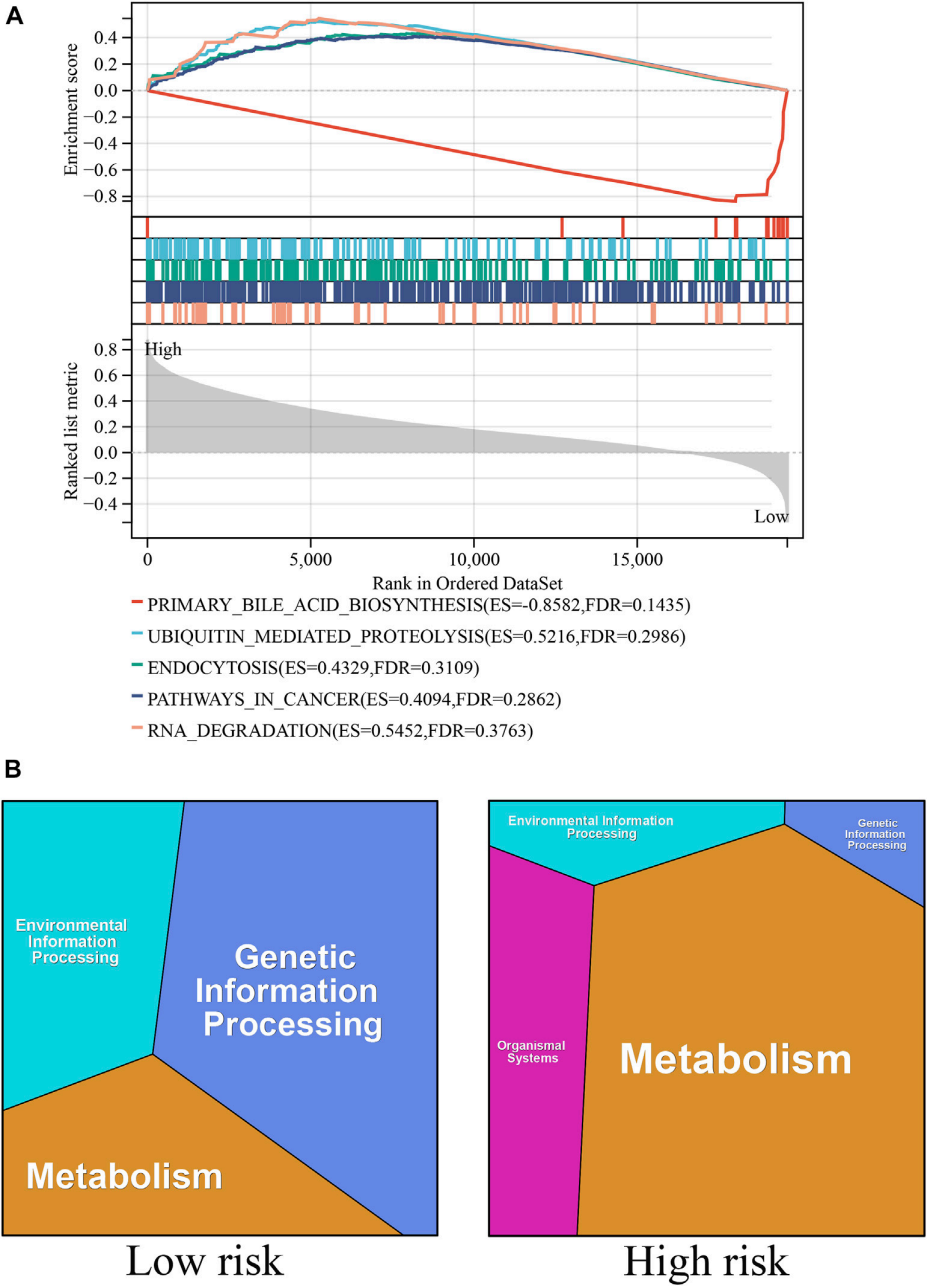
Biological pathways associated with ICD-related genes. **(A)** The GSEA functional analyses in the high- and low-risk groups. **(B)** The KEGG functional analyses in the high- and low-risk groups.

GSEA and KEGG functional analyses were performed to explore the biological mechanisms of the ICD-related genes in the high- and low-risk groups (Figures 5A, B). The top GSEA terms indicated the roles of the ICD-related genes in the regulation of primary bile acid production, ubiquitin-mediated proteolysis, endocytosis, cancer pathway, and RNA degradation. The top KEGG terms indicated the roles of ICD-related genes in the regulation of genetic, metabolic, environmental, and organismal systems.

## Discussion

The treatment and prognosis of liver cancer have been a major concern for physicians. Despite the development of new antitumor medications, the survival rate of patients with liver cancer remains poor. With the development of the ICD concept, an increasing number of researchers have attempted to induce ICD in liver cancer cells using pharmaceuticals in an effort to treat liver cancer, bringing promise to liver cancer treatment. Several studies have demonstrated that the combination of oxaliplatin and immune checkpoint therapy increases the immunological death of liver cancer cells, resulting in favorable outcomes (Zhu et al., 2020). Other studies have also revealed that cabozantinib induces the death of immune cells and has beneficial therapeutic benefits for patients with liver cancer (Scirocchi et al., 2021). Consequently, computer analysis of the differential expression of ICD-related genes in liver cancer and the development of a prognostic model for liver cancer based on the results of the differential analysis is advantageous in liver cancer treatment and prognosis. In this study, we selected three datasets (GSE65372, GSE25097, and TCGA-HCC) from the GEO and TCGA databases, performed differential analysis, and identified five ICD-related genes with differential expression using a Venn diagram. Then, using the TCGA dataset for validation, we built a prognostic model with three prognosis-related genes. The risk model predicted that the prognosis of patients in the low-risk group was favorable relative to that of patients in the high-risk group. We also found that this model accurately predicted the 1-, 3-, and 5-year survival rates of patients with HCC who underwent surgery. Moreover, the distinct infiltration of immune cells in the high- and low-risk groups may be indicative of distinct immunological microenvironments for various risk models. We then validated the model using the immunohistochemistry public database, further showing the model’s usefulness. We expect that the model will have a broader scope for future applications.

We constructed the risk signature using three mRNAs. PRNP serves as an ICD-related gene as well as an autophagy-related gene. Bioinformatics studies have demonstrated that PRNP contributes to the establishment of an HCC-related prognostic signature, which accurately predicts the prognosis of patients with HCC, thereby shedding light on the potential autophagy mechanisms in liver cell cancer (Chen et al., 2021).Kim et al. (2022) found that cellular prion protein encoded by the PRNP gene increases the risk of recurrence and decreases the survival rate of patients with liver cancer after surgery, as was predicted by the model in our study. There have been numerous studies on liver cell cancer and DNM1L. The liver-specific dynamin-related protein 1 (DRP1; gene name: DNM1L) is a key gene that regulates mitochondrial fission. The high expression of DNM1L is indicative of a poor prognosis for patients with HCC. DNM1L overexpression enhances mitochondrial fission in HCC cells, hence promoting the proliferation of HCC cells (Huang et al., 2016). Similarly, studies on mice have indicated that aerobic exercise decreases the expression of DNM1L in liver cell cancer, influences mitochondrial fission, and inhibits the development of liver cell cancer via the PI3K/AKT pathway (Zhao et al., 2021b). Despite the unsatisfactory efficacy of chemotherapy in the treatment of liver cell cancer, studies have demonstrated that inhibiting DNM1L-mediated mitochondrial fission can further promote apoptosis of liver carcinoma cells, thereby providing strong preclinical evidence for the development of mitochondrial autophagy-based combination therapies (Ma et al., 2020). Consistent with our findings, a study discovered that CASP8, a gene associated with pyroptosis, can contribute to the building of a prognostic signature, and the model can be used to predict the survival of patients with liver cell cancer and their response to immunotherapy (Zheng et al., 2021). Similarly, Boege et al. (2017) found that liver cancer cells with low levels of CASP8 expression had lower invasiveness and poor proliferation ability. Resultantly, patients had favorable overall survival performance, which was the same as predicted by the model in our study.

ICD is a specific type of cell death that can interfere with the antitumor functions of the immune system (Ladoire et al., 2016; Deng et al., 2020; Galluzzi et al., 2020). Discovering ICD-related gene biomarkers may be of benefit to patients with HCC. In our study, we developed a risk model based on ICD and predicted patients’ prognoses. The risk signature classified all patients into high- and low-risk groups, and we discovered that the prognosis of the patients in the high-risk group was considerably poorer than that of those in the low-risk group. The GO and GSEA and GO analysis revealed that genetic information processing had the highest impact in the low-risk group, whereas metabolism had the highest impact in the high-risk group. The GSEA revealed that the primary bile acid production pathway was significantly enriched, and this may serve as a theoretical foundation for the development of ICD-related therapeutic strategies. In addition, recent studies (Dong et al., 2022) have demonstrated that the combination of immune checkpoint therapy plus ICD treatment is one of the most successful treatment strategies available. Additionally, we discovered significant differences in immune checkpoint markers such as PD-1 and PD-L1 between the high- and low-risk groups, indicating that immune checkpoint therapy combined with ICD treatment has considerable potential in the development of treatment strategies for liver cell cancer.

Despite major advances in its treatment, the prognosis for liver cancer remains poor due to drug resistance, recurrence, and metastasis. Combination therapy with immune checkpoint inhibitors and vascular endothelial growth factor inhibitors are currently used as first-line treatment for advanced liver cancer. With the development of immune checkpoint inhibitors-based therapies, there is renewed optimism for patients with liver cancer. Due to the dependence of these treatments on the immune milieu of the tumor, it is vital to study the immunological environment of liver cancer to select the most effective treatment (Hao et al., 2021; Oura et al., 2021). Studies have shown that increased levels of infiltrating immune cells in the tumor microenvironment are associated with higher risks (Ding et al., 2022). Consequently, we studied the immunological microenvironment and the infiltration of diverse immune cells. The high-risk group exhibited a greater infiltration of immune cells relative to the low-risk group. A single-cell sequencing study associated elevated levels of Treg cells with liver cell cancer, providing a new direction for the immunological treatment of liver cell cancer based on its immune microenvironment (Zheng et al., 2017). Our data indicate that immune cell infiltration is positively correlated with risk, and the high-risk group had greater immune cell infiltration. This finding may suggest that immunotherapy may be more effective for patients with liver cell cancer who are at high risk for disease progression. Next, we will verify the validity of the three genes through *in vivo* and vitro experiments. More clinical and experimental research are required to increase the generalizability of our survival prediction model in clinical practice in the future.

## Conclusion

A risk model based on the ICD-related genes PRNP, DNM1L, and CASP8 was developed to predict the prognosis of liver cancer. This risk model can also predict the immunotherapy response of patients with liver cancer.

## Data Availability

The original contributions presented in the study are included in the article/Supplementary Material, further inquiries can be directed to the corresponding authors.
