# Supplementary figures and images for: ICD-related risk model predicts the prognosis and immunotherapy response of patients with liver cancer
Source: Front Pharmacol. 2023 Jun 8;14:1202823. doi: 10.3389/fphar.2023.1202823 (PMC10285067; doi:10.3389/fphar.2023.1202823)

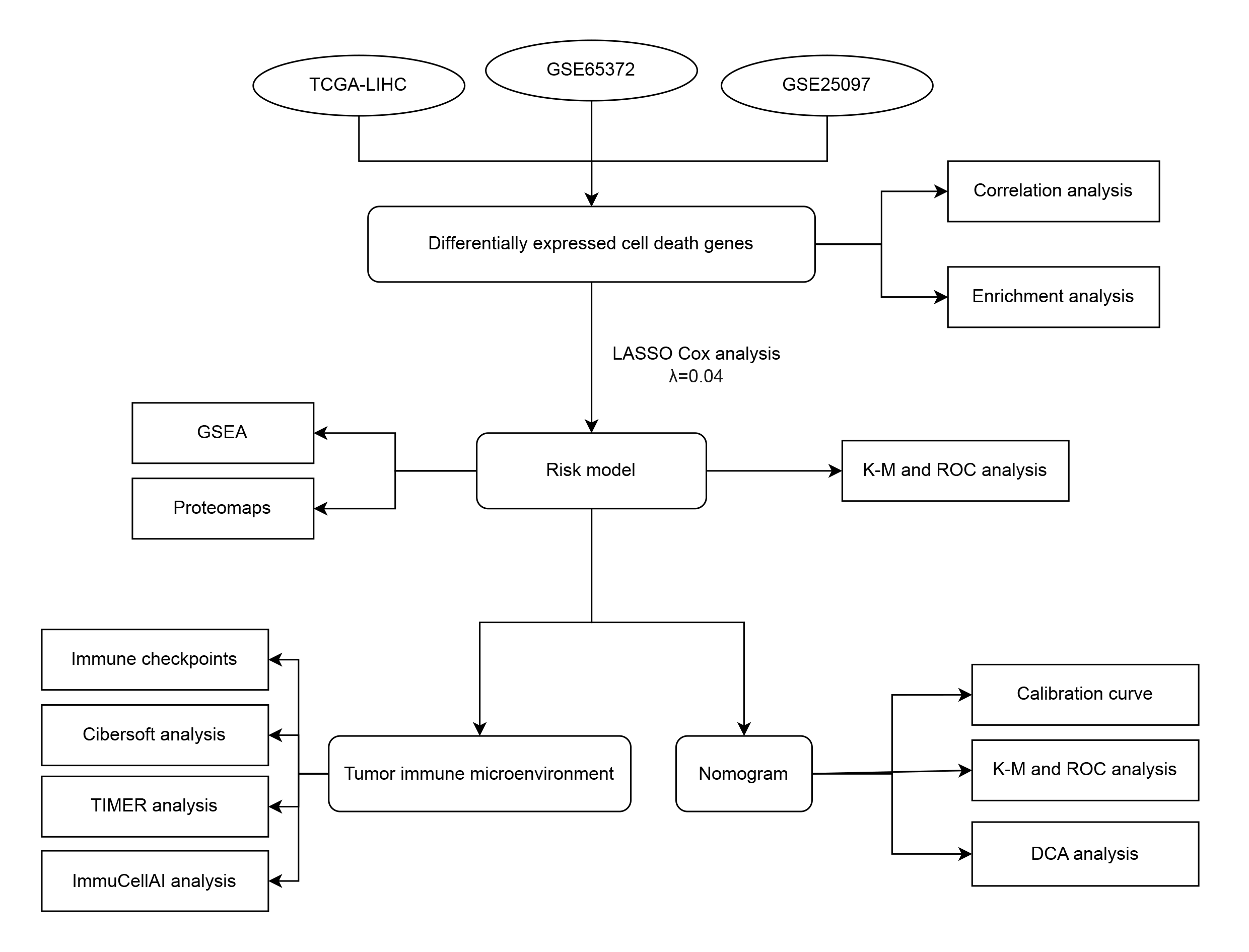

Supplement: Supplementary file 1 [file Image1.TIF]
